# The Physicians' Diary for 1858

**Published:** 1857-12

**Authors:** 


*The Physicians' Diary for* 1858. Buffalo: Phinney & Co., Publishers.

We suppose that there are few of our readers in the city who have not made use of the Diary which we have before us; a visiting list has now become indispensible to every physician, and here we have, in a form even more compact than the mere visiting list of Lindsay & Blakiston, the addition of a medical and obstetrical record sufficiently complete for all practical purposes, namely, columns opposite the name of each patient, for the name of the disease, termination, and remarks; and in the obstetrical record, columns for the presentation (a list of abbreviations being found in the preface) the number of pregnancy, age, sex of child, hours in labor, and description of labor, whether artificial or natural. We mention all these particulars, for from the small and convenient size of this little Diary, one would not expect to find so much space for notes and records. In addition to this, we have space for nurses' addresses, poisons and their antidotes, etc. Though it is often desirable to make a more comprehensive record of cases than it is possible to make in this diary, yet the points which we would notice here, would materially assist in making out a fuller history, and many cases would be sufficiently described, which would otherwise pass with no record at all.

We would cordially recommend this companion to every practitioner of medicine, either in the city or county, believing it to be the best form for a diary which could be adopted. F.

